# Delayed gadolinium-enhanced MRI of meniscus (dGEMRIM) and cartilage (dGEMRIC) in healthy knees and in knees with different stages of meniscus pathology

**DOI:** 10.1186/s12891-016-1244-z

**Published:** 2016-09-29

**Authors:** Ulf Sigurdsson, Gunilla Müller, Carl Siversson, Eveliina Lammentausta, Jonas Svensson, Carl-Johan Tiderius, Leif E. Dahlberg

**Affiliations:** 1 Department of Orthopaedics, Lund University, Skåne University Hospital, SE-205 02 Malmö, Sweden; 2 Institute of Radiology und Scintigraphy, Kantonsspital Lucerne, Spitalstrasse, 6000 Lucerne, Switzerland; 3 Medical Radiation Physics, Department of Translational Medicine, Lund University, Skåne University Hospital, SE-205 02 Malmö, Sweden; 4 Department of Diagnostic Radiology, Oulu University Hospital, PO Box 50, FI-90029 OYS Oulu, Finland; 5 Medical Imaging and Physiology, Skåne University Hospital, SE-221 85 Lund, Sweden; 6 Department of Orthopaedics, Clinical Sciences Lund, Lund University, Skåne University Hospital, SE-221 85 Lund, Sweden

**Keywords:** dGEMRIC, dGEMRIM, Glycosaminoglycans, Meniscus, Cartilage

## Abstract

**Background:**

Lesions in the meniscus are risk factors for developing knee osteoarthritis (OA), not least because of the role of the meniscus in the pathological progression of OA. Delayed gadolinium enhanced MRI of cartilage (dGEMRIC) has extensively been used to identify pre-radiographic cartilage changes in OA. In contrast, its counterpart with regard to examination of the meniscus, gadolinium enhanced MRI of meniscus (dGEMRIM), has been less utilized. In this study we use 3D dGEMRIM in patients with meniscus lesions and compare them with previous results of healthy individuals.

**Methods:**

Eighteen subjects with MRI-verified posteromedial meniscus lesions and 12 healthy subjects with non-injured and non-symptomatic knee joints, together 30 volunteers, were examined using 3D Look-Locker sequence after intravenous injection of Gd-DTPA^2−^ (0.2 mmol/kg body weight). Relaxation time (T1) was measured in the posterior meniscus and femoral cartilage before and 60, 90, 120 and 180 min after injection. Relaxation rate (R1 = 1/T1) and change in relaxation rate (ΔR1) were calculated. For statistical analyses, Student’s *t*-test and Analysis of Variance (ANOVA) were used.

**Results:**

The pre-contrast diagnostic MRI identified two sub-cohorts in the 18 patients with regard to meniscus injury: 1) 11 subjects with MRI verified pathological intrameniscal changes (grade 2) in the posteromedial meniscus only and no obvious cartilage changes. The lateral meniscus showed no pathology. 2) 7 subjects with MRI verified pathological rupture (grade 3) of the posteromedial meniscus and pathological changes in the lateral meniscus and/or medial and lateral joint cartilage.

Comparisons of pathological and healthy posteromedial meniscus revealed opposite patterns in both T1_Gd_ and ΔR1 values between pathological meniscus grade 2 and grade 3. The concentration of the contrast agent was lower than in healthy meniscus in grade 2 lesions (*p* = 0.046) but tended to increase in grade 3 lesions (*p* = 0.110). Maximum concentration of contrast agent was reached after 180 min in both cartilage and menisci (except for grade 3 menisci where the maximum concentration was reached after 90 min).

**Conclusion:**

dGEMRIM and dGEMRIC may be feasible to combine in vivo, preferably with one examination before and one 2 h after contrast injection. Possible different dGEMRIM patterns at different stages of meniscus lesions must be taken into account when evaluating meniscus pathology.

## Background

Osteoarthritis (OA) affects approximately 10 % of the elderly and is a leading cause of disability [1]. OA is suggested to be a whole-joint disease involving cartilage, ligaments, periarticular muscles, subchondral bone and menisci [2]. The meniscus has a critical protective role for knee joint integrity by absorbing impact and distributing load [3, 4]. Overall, it is estimated that the knee meniscus carries 45 to 75 % of the total joint load [5]. Loss of meniscal function has been strongly associated with development and progression of radiographic OA [6, 7]. However, OA-related changes observed by radiography are late events in the degenerative process [8, 9]. In OA, the cartilage matrix undergoes pathological molecular cleavage with loss of type II collagen and glycosaminoglycans (GAG) [1, 10]. To increase our understanding of pathogenic mechanisms in the early stage of the disease, anterior cruciate ligament and meniscus injured patients, with increased risk for OA, may serve as models [6, 11]. Delayed gadolinium enhanced magnetic resonance imaging of cartilage (dGEMRIC) is an imaging technique that can identify pre-radiographic degenerative changes in articular cartilage, and potentially also in the meniscus [12, 13]. When this technique is applied to the latter tissue, it is designated gadolinium enhanced MRI of meniscus (dGEMRIM). In dGEMRIC, the fixed charged density of GAG is studied in vivo through quantitative measurement of the longitudinal relaxation time (T1) of the articular cartilage in the presence of the negatively charged contrast agent Gd-DTPA^2−^ (gadolinium diethylene triaminepentaacetic acid). Recently, we have shown in healthy subjects that the contrast medium distributes also into the meniscus after intravenous injection (dGEMRIM) [13]. The purpose of our study is to evaluate dGEMRIM as a diagnostic tool in menisci with MRI verified pathological changes. The literature has indicated conflicting results concerning the GAG content in pathological meniscus [14–18]. Since these results may differ depending on the grade of pathological changes we have two separate main hypotheses. Firstly, we assume that meniscus with pathological grade 2 changes will display values suggesting an increased content of GAG compared to healthy meniscus and secondly, we assume that meniscus with pathological grade 3 changes will display values suggesting a decreased content of GAG compared to healthy meniscus. The specific aims of the study were first to investigate whether there is a measurable uptake of contrast agent in pathological menisci and when the maximum level of contrast agent was obtained, as measured in minutes after contrast injection. Second, we investigate whether there is a difference between the uptake of contrast agent in the posteromedial pathological meniscus and the posteromedial healthy meniscus in a previously examined knee without symptoms.

## Methods

### Subjects

In our study, we analyzed contrast enhanced MRI images of one knee in each of 30 volunteers. 12 of them were included in a prior study of contrast-enhanced MRI of healthy articular cartilage and meniscus of the knee [13]. Inclusion criteria for these 12 subjects (5 males, age 23–28 years, mean 25 years) were: 1) no history of injury or pain of the knee; 2) no abnormality at physical examination of the knee; 3) no pathologic changes at diagnostic MRI. 18 subjects with knee pain (experienced for a period of more than two months) were recruited by reviewing medical records. The knee pain was either due to spontaneous appearance or to a minor pivoting trauma and they had an MRI-verified posteromedial meniscus injury.

Exclusion criteria for all 30 subjects were: 1) contraindications for MRI (i.e. metal prosthesis, claustrophobia); 2) history of previous reactions to contrast agent and 3) medical record of kidney pathology.

### Contrast enhanced MRI

The contrast enhanced MRI examination was performed within 4–6 months from the first diagnostic MRI. Prior to the intravenous injection of Gd-DTPA^2−^ (Magnevist®, Bayer Schering Pharma Ag, Berlin, Germany), a diagnostic and quantitative T1 measurement examination was performed in the 18 subjects with injured meniscus. Meniscus changes were graded according to the classification of Lotysch [19]. Grade 1 changes represent one or several punctate signal intensities at one slice (3 mm between the slices), grade 2 changes represent a linear (i.e. observed in several slices) intrameniscal signal intensity without extension to the articular surface and grade 3 changes represent a signal intensity extended to at least one articular surface.

Double dose of Gd-DTPA^2−^ (0.2 mmol/kg body weight) was given in an antecubital vein. The time-point zero was set at the end of the drug injection. After injection, the subjects walked for ten minutes to optimize the distribution of Gd-DTPA^2−^ into the meniscus and cartilage. Subsequent examinations were performed at four time points (60, 90, 120 and 180 min) after the injection. Data was collected using a 1.5 T MRI scanner (Siemens Sonata) with a dedicated knee coil. A three dimensional (3D) Look-Locker sequence (field of view (FOV) 160 × 160, Matrix 256 × 256, 30 slices, slice thickness: 3 mm, repetition time (TR) 2500 ms, Flip Angle 6°, 10 inversion times (TIs) was used to acquire all 3D T1 maps. T1was calculated using the Pre-calculated Flip Angle Correction method and the associated flip angle slab profile was acquired from previous phantom measurements [20]. All data were evaluated using software programmed in MATLAB (The MathWorks Inc., Natick, MA, USA).

**Fig. 1:**
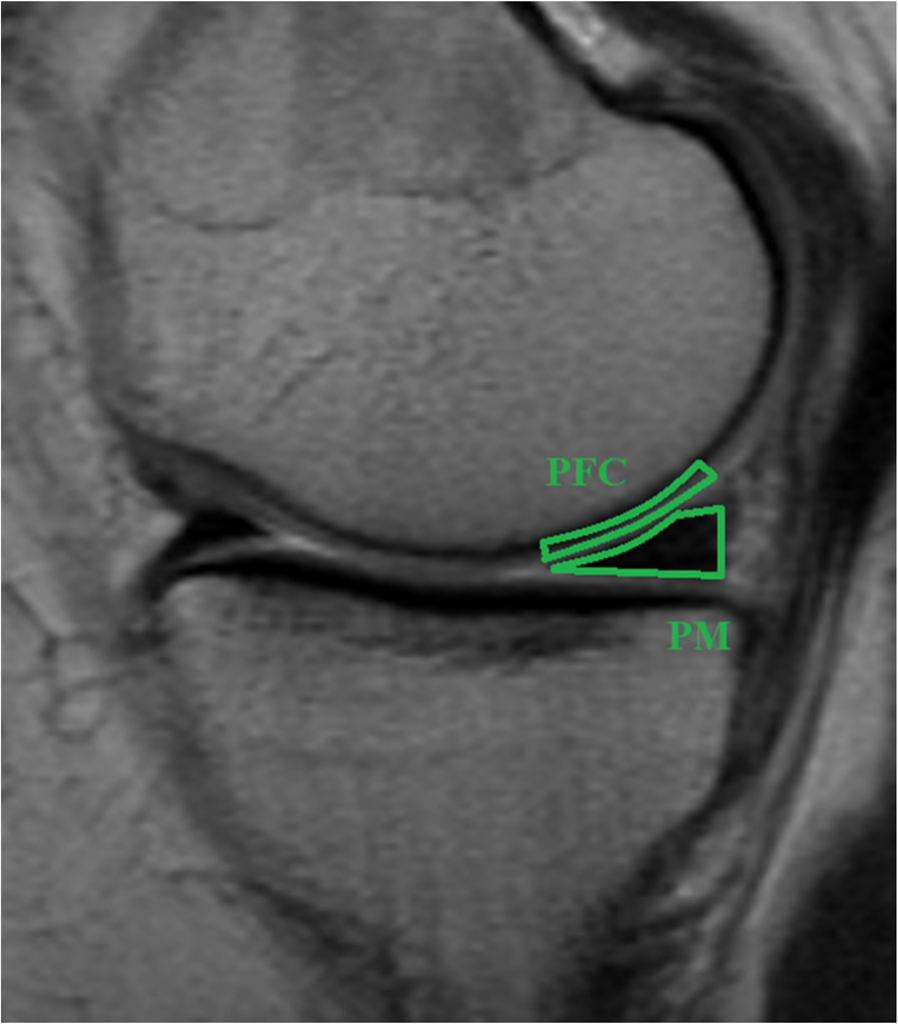
Sagittal view of a knee joint. The average T1 value was calculated in the following regions of interest: PFC (Posterior Femoral Cartilage) and PM (Posterior Meniscus)

For the contrast-enhanced analysis, a radiologist used the pre-contrast MRI examination to define the location of the pathological meniscus changes. From that location, two sagittal slices, one in the lateral and one in the medial compartment, were selected from the 3D volume to enable analysis of the damaged posterior horn of the medial meniscus and the corresponding posterior part of the lateral meniscus. The same slices were used to examine the femoral articular cartilage in order to evaluate the optimal time point for combined analysis of cartilage and meniscus. Four ROIs were drawn in each knee to cover the posterior portion of the medial and lateral meniscus as well as the overlying femoral articular cartilage (Fig. 1) [21]. To standardize the procedure, all ROIs were drawn by a single investigator (US).

An average medial and lateral meniscus and corresponding cartilage pre- and post-contrast (T1_Pre_, T1_Gd_) value was calculated for each of the four ROIs. The ΔR1 value, which reflects the concentration of Gd-DTPA^2−^ in the tissue, was calculated using the formula ΔR1 = 1/T1_Gd_-1/T1_Pre_.

For statistical evaluation Student’s *t*-test (when comparing cohorts at a specific time point) and analysis of variance (ANOVA) (when comparing cohorts over time) were used. The results are presented in the figures as mean values and with 95 % confidence interval (95 % CI). A *p*-value of <0.05 was considered statistically significant.

All the subjects volunteered to participate in the study. All subjects were orally and by letter informed and a written consent was obtained. The study was approved by the ethics review board in Lund, Sweden.

## Results

### Characterization of subjects

The pre-contrast diagnostic MRI identified two sub-cohorts within the 18 patients with meniscus injury. 11 subjects (7 males, age 31–52 years, mean 40 years) had MRI verified pathological intrameniscal changes (grade 2) in the posteromedial meniscus only and no obvious cartilage changes. The lateral meniscus showed no pathology. In the other 7 subjects (5 males, age 36–63 years, mean 54 years) the MRI examination showed a pathological rupture (grade 3) of the posteromedial meniscus and concomitant pathological changes in the lateral meniscus and/or medial and lateral joint cartilage.

### T1_Pre_, T1_Gd_, and ΔR1 values of healthy and pathological posteromedial meniscus

**Fig. 2:**
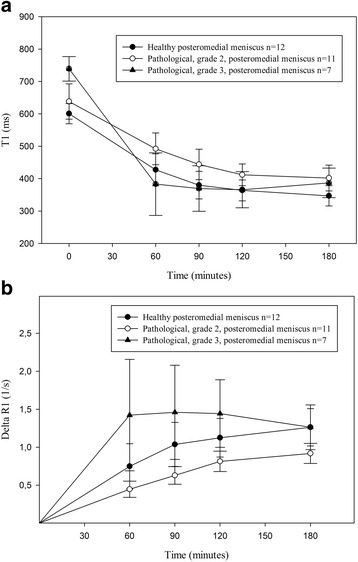
**a** T1_Pre_ and T1_Gd_ values after intravenous injection of Gd-DTPA^2−^ (0.2 mmol/kg body weight), in posteromedial healthy (*n* = 12; closed circles), pathological grade 2 (*n* = 11; open circles) and pathological grade 3 (*n* = 7; closed triangles) menisci. T1_Gd_ was longer in the pathological grade 2 menisci (*p* = 0.027, ANOVA) and showed no difference in the pathological grade 3 menisci compared to the healthy menisci. **b** ΔR1 values in posteromedial healthy (*n* = 12; closed circles), pathological grade 2 (*n* = 11; open circles) and pathological grade 3 (*n* = 7; closed triangles) menisci, after an intravenous injection of Gd-DTPA^2−^ (0.2 mmol/kg body weight). The values were lower in the pathological grade 2 menisci (*p* = 0.046, ANOVA) and tend to show higher values in the pathological grade 3 menisci (*p* = 0.110, ANOVA) compared to the healthy menisci

Subjects with grade 2 lesions showed no difference in T1_Pre_ values compared to subjects with healthy meniscus (*p* = 0.189, *t*-test). However, subjects with grade 3 lesions had longer T1_Pre_ values than subjects with healthy meniscus (*p* < 0.001, *t*-test) (Fig. 2a and Table 1). The contrast medium diffused gradually into the menisci, with a maximum level observed after 180 min. The maximum contrast level for grade 3 menisci was 90 min. Subjects with grade 2 lesions had longer T1_Gd_ values than subjects with healthy meniscus (*p* = 0.027, ANOVA) (Fig. 2a and Table 1). There was no difference in T1_Gd_ in the posteromedial meniscus between subjects with meniscus lesions grade 3 and subjects with healthy meniscus (*p* = 0.315, ANOVA) (Fig. 2a and Table 1).

**Table 1:** T1_Pre_, T1_Gd_ and ΔR1 values in healthy (*n* = 12), pathological grade 2 (*n* = 11) and pathological grade 3 (*n* = 7) posteromedial menisci

T1Pre,T1Gd and ΔR1 values in healthy and pathological- grade 2 and 3- posteromedial meniscus															
Time (min)	Precontrast (Mean(CI) T1Pre (ms))			60			90			120			180		
	Healthy	Grade 2	Grade 3	Healthy	Grade 2	Grade 3	Healthy	Grade 2	Grade 3	Healthy	Grade 2	Grade 3	Healthy	Grade 2	Grade 3
Mean(CI) T1Gd (ms)	601 ± 31	638 ± 54	739 ± 38	427 ± 49	492 ± 49	383 ± 96	379 ± 43	444 ± 46	370 ± 70	364 ± 32	412 ± 34	366 ± 55	347 ± 31	402 ± 40	387 ± 45
*p* values		0,189	<0,001		0,052	0,307		0,036	0,761		0,032	0,936		0,024	0,106
% of difference		6	23		15	11		17	<1		13	<1		16	12
Mean(CI) ΔR1 (1/s)				0,75 ± 0,30	0,47 ± 0,17	1,42 ± 0,73	1,04 ± 0,29	0,70 ± 0,18	1,46 ± 0,62	1,13 ± 0,25	0,86 ± 0,20	1,44 ± 0,44	1,26 ± 0,25	0,93 ± 0,22	1,26 ± 0,30
*p* values					0,099	0,031		0,044	0,120		0,089	0,138		0,041	0,997
% of difference					60	89		49	37		31	27		35	<1

The concentration of the contrast medium, reflected by ΔR1, was lower in meniscus with grade 2 lesion than in healthy meniscus (*p* = 0.046, ANOVA). Meniscus with grade 3 lesions tend to have higher concentration compared to meniscus in subjects with healthy knee joints (*p* = 0.110, ANOVA) (Fig. 2b and Table 1).

### Temporal and spatial contrast distribution patterns of T1_Pre_, T1_Gd,_ and ΔR1 values in posterior articular cartilage

The maximum level of contrast medium in the femoral cartilage was observed after 120–180 min (data not shown). Within knees with a medial meniscus lesion grade 2, mean T1_Pre_, T1_Gd,_ and ΔR1 did not differ in medial and lateral femoral cartilage. Further, no difference in T1_Pre_, T1_Gd,_ and ΔR1 values was found in lateral femoral cartilage in subjects with a medial meniscus injury grade 2 and subjects with healthy knee joints (data not shown). In the medial femoral cartilage, T1_Gd_, but not T1_Pre_ or ΔR1, was longer in subjects with meniscus injury grade 2 compared to subjects with healthy knee joints (*p* = 0.027, ANOVA, *p* = 0.159, *t*-test and *p* = 0.280, ANOVA). The compartments with grade 3 lesions had cartilage changes that were severe enough to compromise the T1_Gd_ calculations. These results were therefore not evaluated.

## Discussion

In our study we show that dGEMRIM can differentiate between pathological (grade 2) and healthy meniscal tissue. The decreased distribution of Gd-DPTA^2−^ in the pathological menisci grade 2 suggests more negative charge and an increased GAG concentration in this meniscus pathology. These results are consistent with previous meniscus studies that showed increased GAG concentration by histology and biochemical analysis [14, 15]. In addition, it has also been reported that proteoglycan content (μg/mg dry weight) in menisci with meniscal tears increase in relation to the severity of the meniscal degeneration [16]. Interestingly, the proteoglycan content in the meniscus does not seem to increase in rheumatoid arthritis [22].

A previous dGEMRIM study of 17 subjects with knee symptoms and MRI verified pathology in cartilage and menisci, showed a trend towards decreased T1_Gd90_ values (i.e. values obtained 90 min after injection of contrast agent) in pathologically changed meniscus (posteromedial grade 2; *n* = 5) compared to healthy meniscus within the same knee joint [17]. All patients in that study were diagnosed with OA (Kellgren & Lawrence grade 1 or 2) and the results are in agreement with the results for the meniscus tissue with pathological changes grade 3 in our study. ∆R1 values, not presented in the study by van Tiel et al., may have enabled a more thorough comparison with our study. It can be speculated that increased GAG synthesis, reflected by longer T1_Gd_ and decreased ΔR1 values for pathological meniscus grade 2 in knee joints without cartilage damages in our study, is an early event in meniscus pathology. In contrast, in later stages of degenerative disease as presented in our study by pathological meniscus grade 3 and in the study by van Tiel et al. (knee joints with meniscus changes grade 2 and diagnosed OA, Kellgren & Lawrence grade 1 or 2), a more extensive damage to the knee joint results in a facilitated diffusion of contrast medium into the meniscus with corresponding shorter T1_Gd_ and increased ΔR1.

In our study the maximum level of contrast medium in healthy femoral cartilage occurred approximately 120 min after injection. This is consistent with earlier results [23].

Comparing the femoral cartilage between healthy knee joints and knee joints with pathological grade 2 changes in the posteromedial meniscus, we found no differences with regard to the T1_Pre_, T1_Gd_ and ΔR1 values on the lateral side. On the medial side, there was a difference in T1_Gd_ but not in the T1_Pre_ or ΔR1 values. These findings strengthen the results from the diagnostic MRI, suggesting that the posteromedial meniscus is the only tissue in the examined knee joint with pathological meniscus changes grade 2 that has pathological changes.

It is important to acknowledge that other factors than GAG content may contribute to the distribution of contrast agent into a specific tissue. The fact that the wet weight GAG concentration in the meniscus is 0.3 % as opposed to 2.0 % in articular cartilage [24] and that collagen network differs between the two tissues [25, 26], suggests that GAG content is not the only factor that determines the contrast distribution into the meniscus. Previous studies [18, 27] addressed this issue in more detail using both an ionic (inversely related to the GAG content) and a non-ionic (no known interaction with GAG content) contrast agent when investigating meniscus and femoral cartilage in knee joints with OA. Data from these studies demonstrated similar differences in T1_Gd_ values in meniscus and cartilage when comparing subjects with OA and healthy volunteers, with both kinds of contrast agents, indicating that GAG is not the sole factor influencing the uptake of contrast agent. The authors of these studies speculated that the integrity of the collagen network may play a role, as well as increased diffusion due to degenerative changes of both the meniscus and cartilage.

Using dGEMRIC, Hawezi et al. [28] showed a depth-wise variation in contrast distribution in femoral knee cartilage. The maximum concentration of the contrast agent was observed at different time points in different cartilage layers: at 120–180 min in the superficial layer and at 240 min or more in the deeper layers. Furthermore, the differences increased with cartilage thickness. Although the GAG content and the composition of different collagen types differ when comparing different parts of the meniscus [23–25], it is unlikely that there is a larger depth-wise alteration of the concentration of the contrast agent in the meniscus since the contrast agent diffuses into the meniscus from two sides. These authors further discussed a wash out effect of contrast agent occurring after approximately 120 min. It has previously been demonstrated that following an intravenous Gd-DTPA^2−^ injection, the gadolinium concentration in blood plasma will reach its maximum and then decrease within 120 min [29]. Assuming similar concentration decay in synovial fluid, the gadolinium concentration of articular cartilage will be higher than that in synovial fluid and a wash out of gadolinium from the cartilage will start approximately after 120 min. The wash out effect needs to be considered in order to choose an optimal time point post contrast to investigate pathological menisci with dGEMRIM. From our study as well as previous studies, it can be concluded that 120 min post contrast seems optimal for the evaluation of both meniscus and cartilage tissue [13].

It may be argued that the exclusion of grade 1 meniscus lesions is a limitation of the present study. However, grade 1 changes are very difficult to depict when drawing ROIs on a specific slice. It is therefore a significant risk that the measurements would be calculated on the wrong slice and resulting in incorrect values.

The results of menisci with pathological changes grade 3 may be an issue for discussion. Firstly, these changes are often considered as an advanced stage of OA and an examination with contrast enhanced MRI would in clinical use be of less interest. Furthermore, meniscal tears might fill with contrast agent directly from joint cavity, causing erroneously T1_Gd_ relaxation times. Therefore, this must be taken in consideration when evaluating the obtained values using dGEMRIM to examine menisci with pathological grade 3 changes.

An issue using gadolinium as contrast agent is the possible occurrence of nephrogenic systemic fibrosis (NSF). This has mainly been observed in patients with severe chronic or acute renal failure following exposure to gadolinium-based contrast agents [30]. However, after the introduction of clinical guidelines restricting the use of gadolinium in these patients, reports from several academic medical centers have been published indicating that no new cases of NSF have been observed among this category of patients [31, 32].

## Conclusions

dGEMRIM and dGEMRIC may be feasible to combine in vivo, preferably with one examination before and one 2 h after contrast injection. Possible different dGEMRIM patterns at different stages of meniscus lesions must be taken into account when evaluating meniscus pathology.

## Abbreviations

3D: Three-dimensional
ΔR1: Difference in R1, reflecting the Gd-DTPA^2−^ concentration
ANOVA: Analysis of variance
CI: Confidence interval
dGEMRIC: delayed gadolinium-enhanced magnetic resonance imaging of cartilage
dGEMRIM: delayed gadolinium-enhanced magnetic resonance imaging of meniscus
FOV: Field of view
Gd-DTPA^2−^: Gadolinium diethylene triaminepentaacetic acid
GAG: Glycosaminoglycan
MRI: Magnetic resonance imaging
NSF: Nephrogenic systemic fibrosis
OA: Osteoarthritis
R1: Relaxation rate, reflecting the Gd-DTPA^2−^ distribution
ROI: Region of interest
T1: Longitudinal relaxation time
T1_pre_: T1 relaxation time without Gd-DTPA^2−^ (pre-contrast)
T1_Gd_: T1 relaxation time after saturation with Gd-DTPA^2−^ (post-contrast)
TI: Inversion time
TR: Repetition time
